# Effect of Inflammation on Gingival Mesenchymal Stem/Progenitor Cells' Proliferation and Migration through Microperforated Membranes: An In Vitro Study

**DOI:** 10.1155/2020/5373418

**Published:** 2020-02-21

**Authors:** M. Al Bahrawy, K. Ghaffar, A. Gamal, K. El-Sayed, V. Iacono

**Affiliations:** ^1^Faculty of Oral and Dental Medicine, Ain Shams University, Cairo, Egypt; ^2^Oral Medicine and Periodontology Department, Faculty of Dentistry, Cairo University, Egypt; ^3^Clinic for Conservative Dentistry and Periodontology, Christian Albrechts University of Kiel, Germany; ^4^School of Dentistry, Stony Brook University, NY, USA

## Abstract

**Background:**

In the field of periodontal guided tissue regeneration, microperforated membranes have recently proved to be very promising periodontal regenerative tissue engineering tools. Regenerative periodontal approaches, employing gingival mesenchymal stem/progenitor cells in combination with these novel membranes, would occur mostly in inflamed microenvironmental conditions intraorally. This in turn entails the investigation into how inflammation would affect the proliferation as well as the migration dynamics of gingival mesenchymal stem/progenitor cells. *Materials and Methods*. Clones of human gingival mesenchymal stem/progenitor cells (GMSCs) from inflamed gingival tissues were characterized for stem/progenitor cells' characteristics and compared to clones of healthy human GMSCs (*n* = 3), to be subsequently seeded on perforated collagen-coated poly-tetra-floro-ethylene (PTFE) membranes with a pore size 0.4 and 3 microns and polycarbonic acid membranes of 8 microns pore size in Transwell systems. The population doubling time and the MTT test of both populations were determined. Fetal bovine serum (FBS) was used as a chemoattractant in the culturing systems, and both groups were compared to their negative controls without FBS. Following 24 hours of incubation period, migrating cells were determined on the undersurface of microperforated membranes and the membrane-seeded cells were examined by scanning electron microscopy.

**Results:**

GMSCs demonstrated all predefined stem/progenitor cell characteristics. GMSCs from inflamed gingival tissues showed significantly shorter population doubling times. GMSCs of inflamed and healthy tissues did not show significant differences in their migration abilities towards the chemoattractant, with no cellular migration observed in the absence of FBS. GMSCs from healthy gingival tissue migrated significantly better through larger micropores (8 microns). Scanning electron microscopic images proved the migratory activity of the cells through the membrane pores.

**Conclusions:**

Inflammation appears to boost the proliferative abilities of GMSCs. In terms of migration through membrane pores, GMSCs from healthy as well as inflamed gingival tissues do not demonstrate a difference in their migration abilities through smaller pore sizes, whereas GMSCs from healthy gingival tissues appear to migrate significantly better through larger micropores.

## 1. Introduction

Periodontitis is an inflammatory degenerative disease associated with bacterial dysbiosis, leading if untreated to progressive loss of tooth-supporting tissues [1, 2]. Gingival mesenchymal stem/progenitor cells (GMSCs) exhibit multipotent differentiation capacities [3] and the potential for complete periodontal regeneration [3–5]. They further play a pivotal role in modulating the inflammatory response in their surrounding microenvironments [6–8].

Melcher was the first to describe guided tissue regeneration (GTR), with a promise for a complete regeneration of the periodontal apparatus [9]. Gamal and Iacono compared a traditional occlusive barrier membrane (OM) used in GTR to a perforated collagen membrane, concluding that the latter was associated with superior clinical outcomes [10]. Recently, the ability of GMSCs from healthy gingival tissue origin to migrate selectively through microperforated membranes with suitable pore size in the presence of chemoattractants was clearly demonstrated [1]. The prospect of developing selective guided tissue regeneration membranes, allowing stem/progenitor cells to migrate through them, while being occlusive to unwanted cell lines, namely, epithelial and fibrous connective tissue cells, would represent a promising tool in the field of tissue engineering-mediated periodontal regeneration.

The objective of the present study was to determine and compare the potential of GMSCs extracted from healthy and inflamed gingival tissues to proliferate and migrate through novel microperforated membranes in vitro, additionally exploring role of the classical FBS chemoattractant factor in this process.

## 2. Materials and Methods

### 2.1. Sample Selection

Gingival connective tissue samples were extracted from discarded gingival specimens of patients with healthy and inflamed gingiva at the periodontal care clinic of Stony Brook University in Long Island, NY, in the course of regular periodontal therapy. Four subjects were included in this study, two for the healthy gingival tissue specimens and two for the inflamed ones. Experiments for each group were done in triplicate (*n* = 3). Informed consent was obtained from all the participants. The study was approved by the Committee of Research Involving Human Subjects at Stony Brook University and Ain Shams University scientific ethical committee (IRB number 575741).

### 2.2. Establishment of Cell Cultures from the Gingival Tissues

Gingival tissue samples were sliced and digested in 2 mg/ml Dispase II (Sigma-Aldrich, St. Louis, USA) at 4°C overnight, followed by 2 mg/ml collagenase IV (Thermo Fisher Scientific, Massachusetts, USA) for 40 minutes at 4°C. The resultant cellular suspension was filtered through a 40 *μ*m cell strainer and centrifuged for 10 minutes at 1200 rpm. Single-cell suspensions were subsequently plated at a concentration of 60 cells/cm^2^ in 10 cm tissue culture dishes for the isolation of single-cell-derived colonies in alpha minimal essential medium (alpha MEM 1×, Gibco) supplemented with 10% fetal bovine serum (FBS; Hyclone, Thermo Fisher Scientific), 50 U/ml penicillin G with 50 *μ*g/ml streptomycin, and 2.5 *μ*g/ml amphotericin B (fungizone, Thermo Fisher Scientific) in a humidified atmosphere (37°C, 5% CO_2_). Cells were subcultured in P100 dishes for further passages. P10 plates were used for the colony-forming unit (CFU) assay.

### 2.3. Population Doubling Time Assay

Population doubling time was determined as previously described [11]. Briefly, GMSCs were seeded at 5 × 10^3^ cells/cm^2^ in 24-well plates, expanded to approximately 90% confluence, detached with 0.05% trypsin/EDTA, and counted. Subsequently, GMSCs were reseeded at 5 × 10^3^ cells/cm^2^ into another 24-well plate and cultured until in vitro cellular senescence was noted. Cells were counted at each passage and population doubling times were calculated using the following formula:

(1) $$\begin{array}{c} \frac{\log2\text{ final cell number}}{\log2\text{ seeding cell number}}. \end{array}$$

Finally, the population doubling time value for the GMSC populations was calculated.

### 2.4. Flow Cytometry Expression of MSC-Associated Markers

GMSCs from the fourth and fifth passages were washed with PBS twice, detached with 0.05% trypsin/EDTA, and resuspended in blocking buffer 1% bovine serum albumin for half an hour. Approximately 1 × 10^5^ cells were incubated for half an hour at 4°C in 2 *μ*g/ml fluorescein isothiocyanate- (FITC-) conjugated mouse monoclonal antibodies specific for human CD73 and its isotype control (BD Pharmigen, San Jose, California, United States), APC-conjugated mouse monoclonal antibodies for CD90 and its isotype control (BD Pharmigen), Alexa 555 gout anti-mouse for primary unconjugated mouse monoclonal antibodies against CD105 (Dako) and its control Alexa 555 gout anti-mouse without primary mouse antibodies, and PE-conjugated mouse monoclonal antibodies for CD146 and its isotype control (BD Pharmingen). In terms of the hematopoietic markers, mouse monoclonal antibodies against CD14, CD34, CD45, and their isotype controls were used. After washing, centrifugation and resuspension twice, cells were analyzed flow cytometrically.

### 2.5. In Vitro Differentiation Capacity

#### 2.5.1. Osteogenic Differentiation

GMSCs were seeded at 8 × 10^3^ cells per cm^2^ in six-well plates in osteogenic inductive medium (Gibco, Stem Pro), and the medium changed twice per week for 28 days [12] [13]. Subsequently, wells were washed twice with PBS, and the cells were fixed with 4% paraformaldehyde for 60 minutes at room temperature, washed twice with distilled water, stained by 2% Alizarin Red for 45 minutes in the dark, and finally washed four times with distilled water and twice in PBS.

#### 2.5.2. Adipogenic Differentiation

GMSCs were seeded at 8 × 10^3^ per cm^2^ in six-well plates in adipogenic inductive medium (Gibco, Stem Pro), and the medium changed twice per week for 28 days [13, 14]. Subsequently, the wells were washed twice with PBS, and the cells fixed in 4% paraformaldehyde for 60 minutes at room temperature and washed twice in distilled water. After washing with 60% isopropanol for 5 minutes, the formation of lipid-laden fat cells was detected in 24-well plates by staining for 5 minutes with Oil Red O in isopropanol (300 mg oil red in 100 ml isopropanol) diluted in distilled water in a ratio of 3 : 2. Finally, the cultures were washed with tap water and stained with hematoxylin for 1 minute and then washed again with tap water and viewed under the phase-contrast inverted microscope.

#### 2.5.3. Chondrogenic Differentiation

GMSCs were seeded at 8 × 10^3^ per cm^2^ in six-well plates and cultured in chondrogenic inductive medium (Gibco, Stem Pro), and the medium changed twice per week for 28 days. After 28 days, the wells were washed twice with PBS, and the cells fixed in 4% paraformaldehyde for 60 minutes at room temperature. The wells were washed twice with distilled water, and the cells were stained with Alican blue (10 mg in 60 ml ethanol with 40 ml acetic acid) overnight in the dark to stain any formed cartilage glycoproteins blue. The wells were finally destained (120 ml ethanol with 80 ml acetic acid) for 20 minutes and washed twice with PBS, and the cultures examined under the microscope.

### 2.6. MTT Assay

GMSCs were seeded in a spectrophotometer tube with 500 *μ*l alpha MEM (Gibco) and 10% FBS (Hyclone, Fisher Scientific). A cell-free tube was used as a control. The tubes were incubated in a humidified atmosphere (37°C, 5% CO_2_) for a day. 100 *μ*l of MTT was added to the tubes and they were incubated for four hours. The media were aspired, and 1000 *μ*l of DMSO was added to each tube. The spectrophotometer read the absorbance of each sample at 595 nm wavelength.

### 2.7. Migration Assay

#### 2.7.1. Microscopic Perforated Membranes

The cell migration assays were performed in a Transwell chemotaxis chamber with two types of membranes (Corning Life Sciences), namely, 12 mm collagen-coated poly-tetra-floro-ethylene (PTFE) membrane inserts with 0.4 *μ*m and 3 *μ*m pores and 6.5 mm polycarbonate membrane inserts with 8 *μ*m pores. GMSCs were harvested using 0.05% trypsin/EDTA and resuspended in serum-free alpha MEM. 1 × 10^4^ GMSCs were seeded in the upper compartments. The experimental groups received alpha MEM with 10% fetal bovine serum (Hyclon, Fisher Scientific), while in the control group, serum-free alpha MEM was used in the lower compartment. The plates were incubated in a humidified atmosphere (37°C, 5% CO_2_). After 24 hours, the media were aspirated, and the inserts were washed twice in PBS. GMSCs on the upper surface of the membranes were removed with a cotton swab, and the cells that migrated to the lower side were fixed with 4% paraformaldehyde for 2 minutes, washed twice in PBS, permealized by 100% methanol for 20 minutes and stained with crystal violet stain (1% in 80% ethyl alcohol, Sigma-Aldrich). The washing was performed again twice in PBS, and the membranes were visualized under light microscopy at 40x magnification.

#### 2.7.2. Scanning Electron Microscopy

The Transwell membranes were cut off the inserts, fixed in 4% PFA, and left to dry. The membrane specimens were dehydrated in a series of 50%, 70%, 80%, 90%, and 100% ethyl alcohol for 10 minutes for each concentration. Finally, the specimens were left overnight at -80°C in a closed box and examined at the electron microscope.

#### 2.7.3. Statistical Evaluation

**Figure 1:**
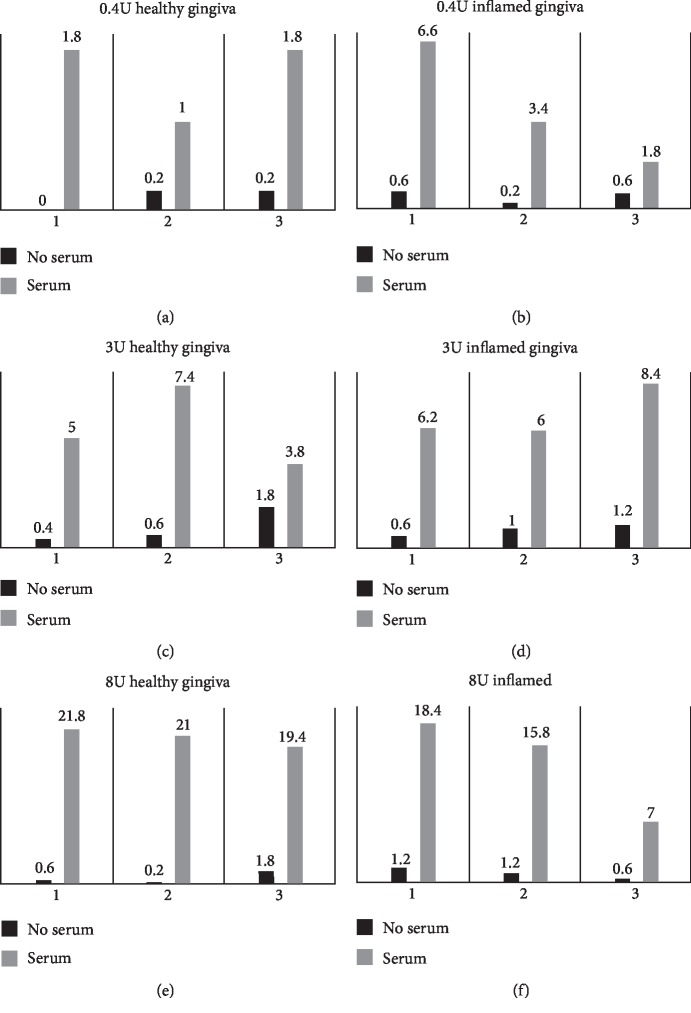
Mean counts of migrated GMSCs isolated from healthy and inflamed gingival tissues through the different pore sizes.

Differences in the outcomes between the groups were done using the Mann–Whitney *U* test (SPSS v20 program, IBM) assuming equal variance and a nonparametric distribution, with value of significance set at *p* < 0.05. Experiments were conducted in triplicates. Graphs were plotted using Microsoft Excel 2007 (Figure 1).

## 3. Results

### 3.1. Colony-Forming Unit Assay

**Figure 2:**
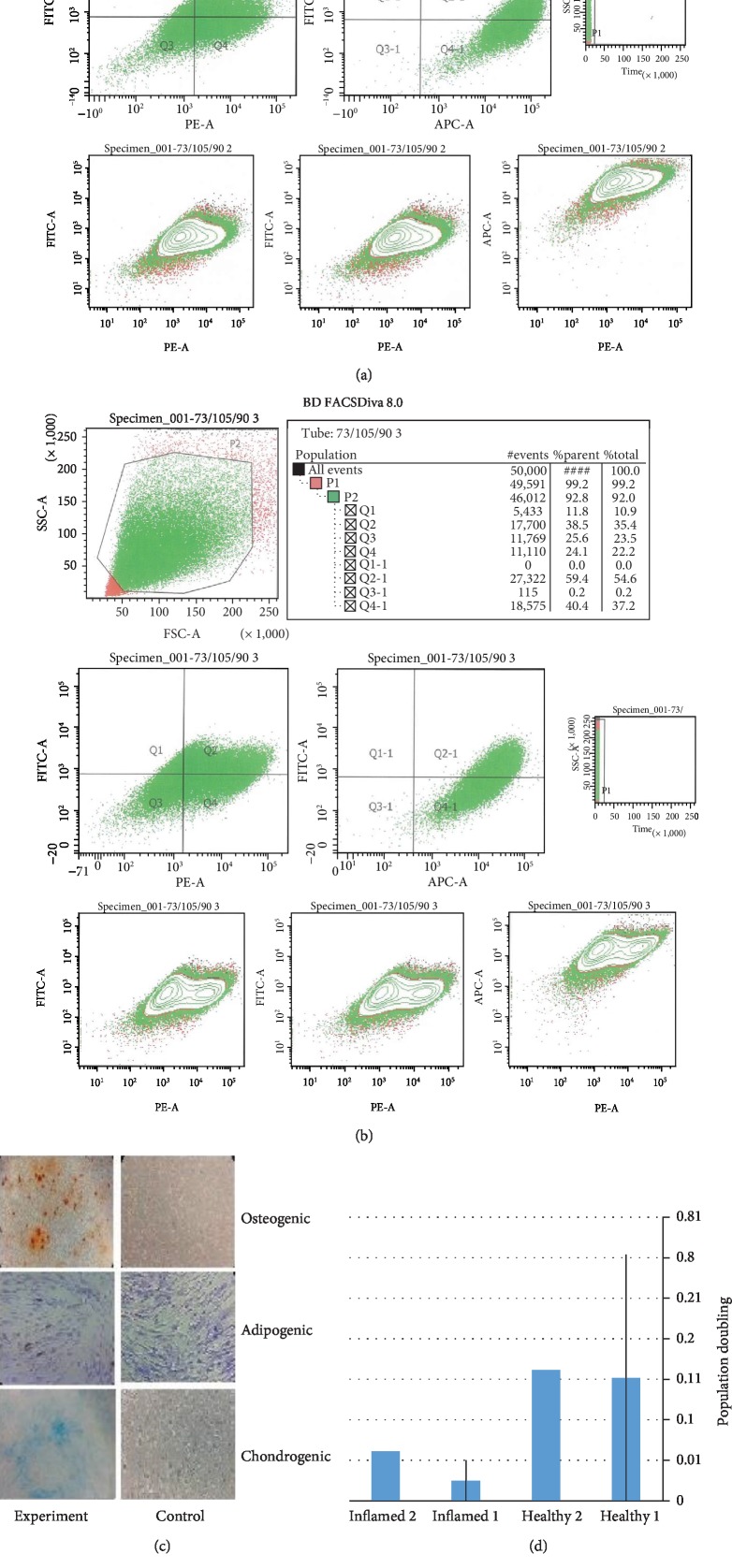
(a) Flow cytometric analysis of the surface marker expressions CD90, CD73, and CD105 in healthy gingival tissues. (b) Flow cytometric analysis of the surface marker expressions CD90, CD73, and CD105 in inflamed gingival tissues. (c) Alizarin red staining of calcium deposits of GMSCs in osteogenic medium, Oil Red staining of oil droplets of GMSCs in adipogenic medium, and Alican blue staining of cartilage glycoprotein of GMSCs in chondrogenic medium and their respective controls. (d) Population doubling time assay with means and standard deviation of the of GMSCs from healthy and inflamed gingival tissues, with significantly shorter population doubling time in the inflamed group. (e) Graph showing colony-forming unit assay with means and standard deviation of the of GMSCs from healthy and inflamed gingival tissues.

Gingival cell suspensions (1000 cells/ml) formed distinctive colonies with typical fibroblastic morphology in P10 dishes after 14 days of culturing in vitro. Experiments for each group were done in triplicates. No significant differences were noted regarding the number of colonies between the healthy and inflamed gingival tissue groups (*p* > 0.05; Mann–Whitney *U* test; Figure 2).

### 3.2. Population Doubling Assay

Both GMSC groups demonstrated remarkable proliferative capacity. The population doubling time was however significantly less in inflamed than in healthy GMSC groups (*p* < 0.05; Mann–Whitney *U* test; Figure 2).

### 3.3. Flow Cytometry Expression of MSC Markers

At passages 4 and 5, cultured GMSCs expressed MSC-associated markers CD105, CD73, CD90, and CD146 and lacked the expression of hematopoietic markers CD14, CD34, and CD45 (Figure 2).

### 3.4. Multilineage Differentiation Capacity

Culturing of GMSCs in osteogenic, chondrogenic, and adipogenic inductive media for 28 days showed remarkable multilineage differentiation ability, which was proved by using Alizarin Red, Alican Blue, and Oil Red, respectively. Using the same stains on the control group grown in 10% serum alpha MEM media did not demonstrate any signs of cellular differentiation (Figure 2).

### 3.5. MTT Assay

The viability and metabolic activities of GMSCs demonstrated no significant differences between experiment and control groups in health and inflamed groups, respectively, using MTT at passage 5 (*p* > 0.05; Mann–Whitney *U* test).

### 3.6. Transwell Migration Assay

#### 3.6.1. Microscopic Perforated Membranes

**Figure 3:**
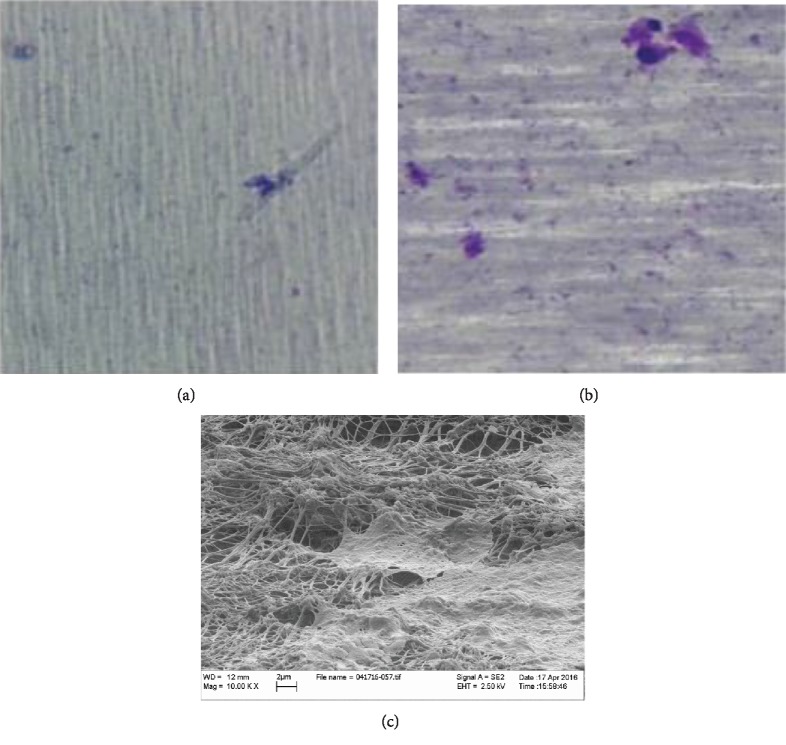
Representative image of migrated GMSCs in lower compartment of 3 microns pores perforated collagen-coated PTFE membranes with GMSCs from (a) inflamed tissues and GMSCs from (b) healthy tissues. (c) SEM image showing migrated GMSCs passing through 3 microns pores of perforated collagen-coated PTFE membranes.

*(1) 0.4 μm and 3 μm Perforated Collagen-Coated PTFE Membranes*. GMSCs significantly migrated through 3 *μ*m and 0.4 *μ*m pores in the chemoattractant as compared to the control group. The migration was lower than the one noted through the membranes with 8 *μ*m pores. No significant difference was found in the migration patterns of the GMSCs isolated from healthy versus inflamed tissues. Comparing the median of the cells from the healthy as well as inflamed clones migrating through the 0.4 *μ*m as well as the 3 *μ*m pores showed no significant differences with a mean rank of 12.6 for the healthy and 18.40 for the inflamed as well as a mean rank of 14.30 for healthy and 16.70 for the inflamed, respectively (*p* > 0.05, Mann–Whitney *U* test; Figure 3).

**Figure 4:**
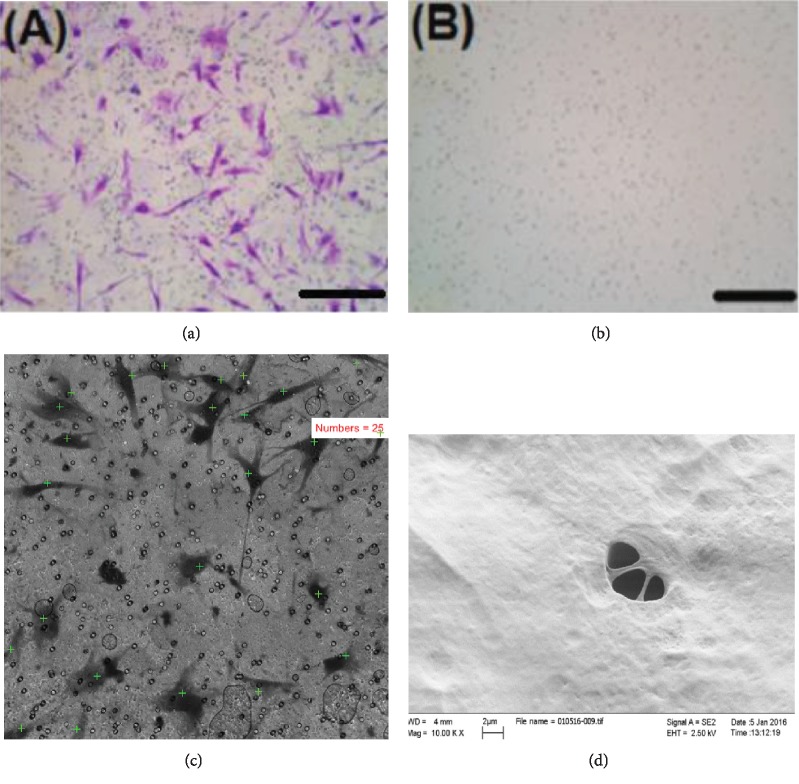
(a) Representative images of 8 *μ*m perforated polycarbonate membranes with migrated cells in the lower compartment of membrane when bovine serum was used as a chemoattractant (cells stained with crystal violet; 10,000 cells seeded in the upper compartment). (b) Lower side of the membrane in the serum-free control group, showing no cells migrated. (c) Cells counted in the lower compartment in 5 random fields, 40x magnification (serum group). (d) SEM image of GMSCs aggregating over 8 microns pores of perforated polycarbonate membrane, with a cell process still inside one of the membrane pores.

*(2) 8 μm Perforated Polycarbonate Membrane*. 40x magnification and flow cytometry assay of the media in the lower compartment could not detect any cells floating in both serum and serum-free groups. Significantly higher migration was notable in favor of GMSCs from healthy gingival tissues as compared to GMSCs from inflamed ones with a mean rank of 19.53 for healthy and 11.47 for the inflamed, respectively (*p* = 0.011; Mann–Whitney *U*; Figure 4).

### 3.7. Scanning Electron Microscopic Examination

No differences were detectable between GMSCs from inflamed and healthy tissues. GMSCs migrating through polycarbonate membrane seemed to look flatter in shape and spread over the membrane, in contrast to cells migrating through the collagen membrane, which looked more bulbous, and confined to the strands of the collagen (Figures 4(d) and 3(c)).

## 4. Discussion

Periodontitis is an inflammatory disorder of the tooth-supporting structures associated with bacterial dysbiosis [15]. In the course of the inflammatory periodontal disease as well as in the initial phases of any periodontal healing, GMSCs interact with their inflammatory microenvironment, affecting their cellular attributes [6]. The present study investigated the proliferative and migratory potentials of GMSCs isolated from healthy and inflamed gingival tissues in the presence and absence of FBS as chemoattractant through membranes with different pore sizes (0.4 *μ*m, 3 *μ*m, and 8 *μ*m) in vitro. The hypothesis was that inflammation would exert an effect on the proliferation and migration of GMSCs.

The investigated GMSCs demonstrated all predefined MSCs' markers, namely, CD105, CD90, and CD73, as well as CD146, CFUs, and a remarkable multilineage differentiation potential into osteoblasts, chondroblast, and adipocytes [8, 14, 16, 17]. Interestingly, in comparison to GMSCs from healthy gingival tissue, GMSCs from inflamed one, similar to earlier investigations [18–21], demonstrated significantly faster proliferation, with a markedly shorter population doubling time.

In the present study, FBS was employed as a chemoattractant to assess the migratory activity of GMSCs through ultrafine pores of the examined membranes. The seeding of 10,000 GMSCs in the upper compartment was deemed suitable to easily identify migrated cells in the lower parts of the membranes. The migration rates in a 24-hour interval varied depending on the pore sizes. A significant difference was demonstrated in the serum-driven migration groups compared to control groups, where GMSCs actively migrated through membrane pores towards the serum in the lower compartment irrespective of pore size, gravity effects, or fluid diffusion.

GMSCs from healthy as well as inflamed tissue origins migrated through the 0.4 *μ*m and 3 *μ*m pores with no significant difference. However, there was a significant difference in cells migration in case of larger 8 *μ*m pores, where cells originating from healthy tissues migrated more actively. This peculiar finding suggests that 8 *μ*m pores might have, despite its larger more permissive diameter, a selective migratory effect on the GMSCs according to their inflammatory status. Furthermore, the observed difference may be attributed to structural characteristics of collagen membranes, with healthy tissue-derived cells sticking more readily to collagen than to polycarbonate, facilitating its migrating through the polycarbonate membrane.

SEM analysis could not determine any morphological differences between the GMSCs derived from healthy and inflamed tissues. GMSCs demonstrated a fibroblast-like morphology under the SEM. However, GMSCs attached on the polycarbonate membranes looked flat and showed more pseudopodia, while the GMSCs adherent on collagen-coated PTFE membranes had a rougher surface and conformed to the shape of collagen strands. The observed morphological differences of the attached GMSCs could be largely attributed to the variability of membrane roughness [22–24] and are consistent with previous investigations on the effect of substrates on the morphology of the attached cells [25, 26] . These findings are further consistent with previous investigations [27], displaying different GMSC morphologies on polycarbonate versus collagen membranes.

## 5. Conclusion

Inflammation of the gingival tissue does not affect the existence of multipotent mesenchymal stem/progenitor cells in them. Although inflammation appeared to boost proliferation as was evident through a shorter population doubling time, regarding the migration dynamics, there was no significant difference in the number of migrated GMSCs through different membrane micropore sizes in the healthy and the inflamed groups, except with large micropore sizes, where GMSCs from healthy tissue demonstrated a higher migratory activity. No migration would occur in the absence of chemoattractant. The present results shed new light on the effect of inflammation and GTR membrane pore size on different attributes of GMSC pivotal for periodontal repair/regeneration and could represent an initial step in the formulation of a novel concept for membrane-driven periodontal guided tissue regeneration.

## Data Availability

The data used to support the findings of this study are available in the study findings, and more details or photos are available from the corresponding author upon request.

## References

[1] Al Bahrawy M., Ghaffar K. A., El-Mofty M., Rahman A. A. (2011). The mutual effect of hyperlipidemia and proinflammatory cytokine related to periodontal infection. *Egyptian Journal of Oral & Maxillofacial Surgery*.

[2] Fawzy El-Sayed K. M., Dörfer C. E. (2017). Animal models for periodontal tissue engineering: a knowledge-generating Process. *Methods*.

[3] Fawzy El-Sayed K. M., Mekhemar M. K., Beck-Broichsitter B. E. (2015). Periodontal regeneration employing gingival margin-derived stem/progenitor cells in conjunction with IL-1ra-hydrogel synthetic extracellular matrix. *Journal of Clinical Periodontology*.

[4] Fawzy El-Sayed K. M., Paris S., Becker S. T. (2012). Periodontal regeneration employing gingival margin-derived stem/progenitor cells: an animal study. *Journal of Clinical Periodontology*.

[5] Wang F., Yu M., Yan X. (2011). Gingiva-derived mesenchymal stem cell-mediated therapeutic approach for bone tissue regeneration. *Stem Cells and Development*.

[6] Fawzy El-Sayed K. M., Elahmady M., Adawi Z. (2019). The periodontal stem/progenitor cell inflammatory-regenerative cross talk: a new perspective. *Journal of Periodontal Research*.

[7] Mekhemar M. K., Adam-Klages S., Kabelitz D., Dorfer C. E., Fawzy El-Sayed K. M. (2018). TLR-induced immunomodulatory cytokine expression by human gingival stem/progenitor cells. *Cellular Immunology*.

[8] Zhang Q., Shi S., Liu Y. (2009). Mesenchymal stem cells derived from human gingiva are capable of immunomodulatory functions and ameliorate inflammation-related tissue destruction in experimental colitis. *The Journal of Immunology*.

[9] Melcher A. H. (1976). On the repair potential of periodontal tissues. *Journal of Periodontology*.

[10] Gamal A. Y., Iacono V. J. (2013). Enhancing guided tissue regeneration of periodontal defects by using a novel perforated barrier membrane. *Journal of Periodontology*.

[11] Menicanin D., Bartold P. M., Zannettino A. C. W., Gronthos S. (2010). Identification of a common gene expression signature associated with immature clonal mesenchymal cell populations derived from bone marrow and dental tissues. *Stem Cells and Development*.

[12] Gronthos S., Graves S. E., Ohta S., Simmons P. J. (1994). The STRO-1+ fraction of adult human bone marrow contains the osteogenic precursors. *Blood*.

[13] Gronthos S., Zannettino A. C. W., Hay S. J. (2003). Molecular and cellular characterisation of highly purified stromal stem cells derived from human bone marrow. *Journal of Cell Science*.

[14] Pittenger M. F., Mackay A. M., Beck S. C. (1999). Multilineage potential of adult human mesenchymal stem cells. *Science*.

[15] Deng Z.-L., Szafranski S. P., Jarek M., Bhuju S., Wagner-Dobler I. (2017). Dysbiosis in chronic periodontitis: key microbial players and interactions with the human host. *Scientific Reports*.

[16] El-Sayed K. M. F., Paris S., Graetz C. (2015). Isolation and characterisation of human gingival margin-derived STRO-1/MACS^+^ and MACS^−^ cell populations. *International Journal of Oral Science*.

[17] Prockop D. J. (1997). Marrow stromal cells as stem cells for nonhematopoietic tissues. *Science*.

[18] Fawzy El-Sayed K., Graetz C., Köhnlein T., Mekhemar M., Dörfer C. (2018). Effect of total sonicated Aggregatibacter actinomycetemcomitans fragments on gingival stem/progenitor cells. *Medicina Oral, Patología Oral y Cirugía Bucal*.

[19] Fawzy El-Sayed K. M., Hein D., Dorfer C. E. (2019). Retinol/inflammation affect stemness and differentiation potential of gingival stem/progenitor cells via Wnt/*β*-catenin. *Journal of Periodontal Research*.

[20] Zhang F., Si M., Wang H., Mekhemar M. K., Dorfer C. E., Fawzy El-Sayed K. M. (2017). IL-1/TNF-*α* inflammatory and anti-inflammatory synchronization affects gingival stem/progenitor cells’ regenerative attributes. *Stem Cells International*.

[21] Zhou L., Dorfer C. E., Chen L., Fawzy El-Sayed K. M. (2017). Porphyromonas gingivalis lipopolysaccharides affect gingival stem/progenitor cells attributes through NF-*κ*B, but not Wnt/*β*-catenin, pathway. *Journal of Clinical Periodontology*.

[22] Brett P. M., Harle J., Salih V. (2004). Roughness response genes in osteoblasts. *Bone*.

[23] Li L., Crosby K., Sawicki M., Shaw L. L., Wang Y. (2012). Effects of surface roughness of hydroxyapatite on cell attachment and proliferation. *Journal of Biotechnology & Biomaterials*.

[24] Ronold H. J., Lyngstadaas S. P., Ellingsen J. E. (2003). Analysing the optimal value for titanium implant roughness in bone attachment using a tensile test. *Biomaterials*.

[25] Huang L., Li R., Liu W. (2014). Dynamic culture of a thermosensitive collagen hydrogel as an extracellular matrix improves the construction of tissue-engineered peripheral nerve. *Neural Regeneration Research*.

[26] Payne J. M., Cobb C. M., Rapley J. W., Killoy W. J., Spencer P. (1996). Migration of human gingival fibroblasts over guided tissue regeneration barrier materials. *Journal of Periodontology*.

[27] Rasmussen C. H., Petersen D. R., Moeller J. B., Hansson M., Dufva M. (2015). Collagen type I improves the differentiation of human embryonic stem cells towards definitive endoderm. *PLoS One*.

